# A Case of Hypersensitivity Pneumonitis with Giant Cells in a Female Dental Technician

**DOI:** 10.1186/2052-4374-25-19

**Published:** 2013-10-04

**Authors:** Yong-Hyun Kim, Yun Kyung Chung, Changhwan Kim, Eun suk Nam, Hyun-Jun Kim, Youngsu Joo

**Affiliations:** 1 Department of Occupational and Environmental Medicine, Hallym University Sacred Heart Hospital, Anyang, Korea; 2 Department of Pulmonary, Allergy and Critical Care Medicine, Kangdong Sacred Heart Hospital, Seoul, Korea; 3 Department of Pathology, Kangdong Sacred Heart Hospital, Seoul, Korea

**Keywords:** Hypersensitivity pneumonitis, Methyl methacrylate, Occupational lung disease, Dental technician, Pneumoconiosis

## Abstract

**Objectives:**

Dental technicians are exposed to methyl methacrylate(MMA) and hard metal dusts while working, and several cases of hypersensitivity pneumonitis caused by the exposure have been reported. The authors experienced a case of hypersensitivity pneumonitis in a female dental technician who had 10 years’ work experience and report the case with clinical evidence.

**Method:**

The patient’s work, personal, social, and past and present medical histories were investigated based on patient questioning and medical records. Furthermore, the workplace conditions and tools and materials the patient worked with were also evaluated. Next, the pathophysiology and risk factors of pneumonitis were studied, and studies on the relationship between hypersensitivity pneumonitis and a dental technician’s exposure to dust were reviewed. Any changes in the clinical course of her disease were noted for evaluation of the work-relatedness of the disease.

**Results:**

The patient complained of cough and sputum for 1 year. In addition, while walking up the stairs, the patient was not able to ascend without resting due to dyspnea. She visited our emergency department due to epistaxis, and secondary hypertension was incidentally suspected. Laboratory tests including serologic, electrolyte, and endocrinologic tests and a simple chest radiograph showed no specific findings, but chest computed tomography revealed a centrilobular ground-glass pattern in both lung fields. A transbronchial biopsy was performed, and bronchoalveolar washing fluid was obtained. Among the findings of the laboratory tests, microcalcification, noncaseating granuloma containing foreign body-type giant cells, and metal particles within macrophages were identified histologically. Based on these results, hypersensitivity pneumonitis was diagnosed. The patient stopped working due to admission, and she completely quit her job within 2 months of restarting work due to reappearance of the symptoms.

**Conclusion:**

In this study, the patient did not have typical radiologic findings, but pathological evaluation of the lung biopsy from the bronchoscope led to the suspicion of pneumonitis. Under the microscope, the sample contained fibrotic changes in the lung, multinucleated giant cells, and particles in macrophages and was diagnosed as dental technician pneumoconiosis by the pathology. Working as a dental technician had directly exposed her to light metal dust and MMA, and her clinical symptoms and radiologic findings subsided after withdrawal from exposure to the workplace. These outcomes led to the diagnosis of hypersensitity pneumonitis due to MMA exposure and strong work-relatedness.

## Background

Hypersensitivity pneumonitis (HP) causes immune associated inflammatory lesions and granulomas in the bronchiole and alveoli of the lungs. It results from repeated aspiration of several causative agents, and it is also commonly called extrinsic allergic alveolitis [1]. Clinically, pneumonitis induces symptoms such as cough and dyspnea, and it manifests in acute, subacute, or chronic forms. In the acute or subacute type, avoiding causative agents usually improves the condition, but repeated and chronic exposure may incite irreversible fibrotic lung disease [1]. Nearly all aspirates, including fungi, bacteria, proteins originating from animals or plants, organic or inorganic chemicals, and metal are known to be causes of HP [2]. A dental technician works in conjunction with a dentist and manufactures, processes, and repairs various dental prostheses and orthodontic devices. In their practice, technicians handle metal alloys, resins, ceramics, plaster, and acrylate, and their job includes casting, grinding, and abrading these materials. They are constantly at risk of exposure to silica, asbestos, methyl methacrylate (MMA), and debris of metal alloys such as chrome, cobalt, molybdenum, nickel, and beryllium. Several case reports, both in Korea and internationally, have been published about occupational lung disease in dental technicians who were exposed to these materials. However, these occupationally acquired lung diseases generally do not show particular symptoms, making the diagnosis difficult. A patient with HP who had worked for 10 years as a dental technician was referred to our department, and we were involved in evaluating and conducting the exposure assessment of the workplace. In addition, the changes in the patient’s clinical progress and exposure level at the workplace were checked serially. The purpose of this report is to describe the findings of work-related HP of a dental technician and the process for determining its work-relatedness.

## Case presentation

### Patient

32-year-old, female.

### Chief complaints and duration

Cough and sputum for 1 year.

### Present illness

The patient complained of intermittent cough and sputum for 1 year, and the patient also had dyspnea while walking upstairs, needing rest midway. For the symptoms, the patient had visited a local otolaryngologist occasionally and had received symptomatic treatments only. She visited our emergency room due to the sudden development of epistaxis. At the time, her systolic blood pressure was measured to be 190 mmHg, but no other specific findings were noted. She was discharged after epistaxis treatment with the recommendation to visit the outpatient clinic. Her blood pressure was again high at 170/120 mmHg, and admission to the department of cardiology was proposed to the patient. After admission, the blood pressures measured on both upper and lower extremities differed by more than 30 mmHg, and chest computed tomography (CT) was taken. On the CT, both lungs showed multiple centrilobular nodules. The patient was again referred to the pulmonary division for additional examinations. Bronchoalveolar lavage (BAL) and transbronchial lung biopsy were performed. Histologically, multinucleated giant cells were observed, coinciding with the findings of HP, and the pathology suggested dental technician’s pneumoconiosis. Thus, the pulmonologist referred the patient to the Department of Occupational and Environmental Medicine to evaluate any specific relationship of the disease with her occupation.

### Past and personal histories

The patient had suffered from cough, sputum, and dyspnea when walking upstairs for 1 year. The patient had not been very concerned about her symptoms at the time, and she only looked for medical treatment when these symptoms had been aggravated. She visited a local otolaryngologist intermittently and was told that her vocal cords showed inflammatory changes and nodules. After taking oral medications, her symptoms improved. She had experienced several episodes of repeated aggravation and relapse of the symptoms. Her symptoms started to aggravate 10 days before admission to our emergency department, With the exception of a 130 mmHg systolic blood pressure measured during a routine health examination 2 years earlier, her past history was insignificant. She did not consume any alcohol or tobacco. Her height was 156 cm. She weighed 70 kg, and recalled no recent loss of weight. She was residing in a apartment building, and questioning about her residence, hobbies, and other activities revealed that she was not exposed to any risk of respiration of dust.

### Physical examination

The blood pressure, pulse, respiratory rate, and temperature checked at the admission were 160/100 mmHg, 76 beats/minute, 19 respirations/minute, and 36.9°C, respectively. She had a consistent cough and intermittent expectoration of white-yellowish sputum. Other findings were unremarkable.

### Laboratory findings

**Figure 1:**
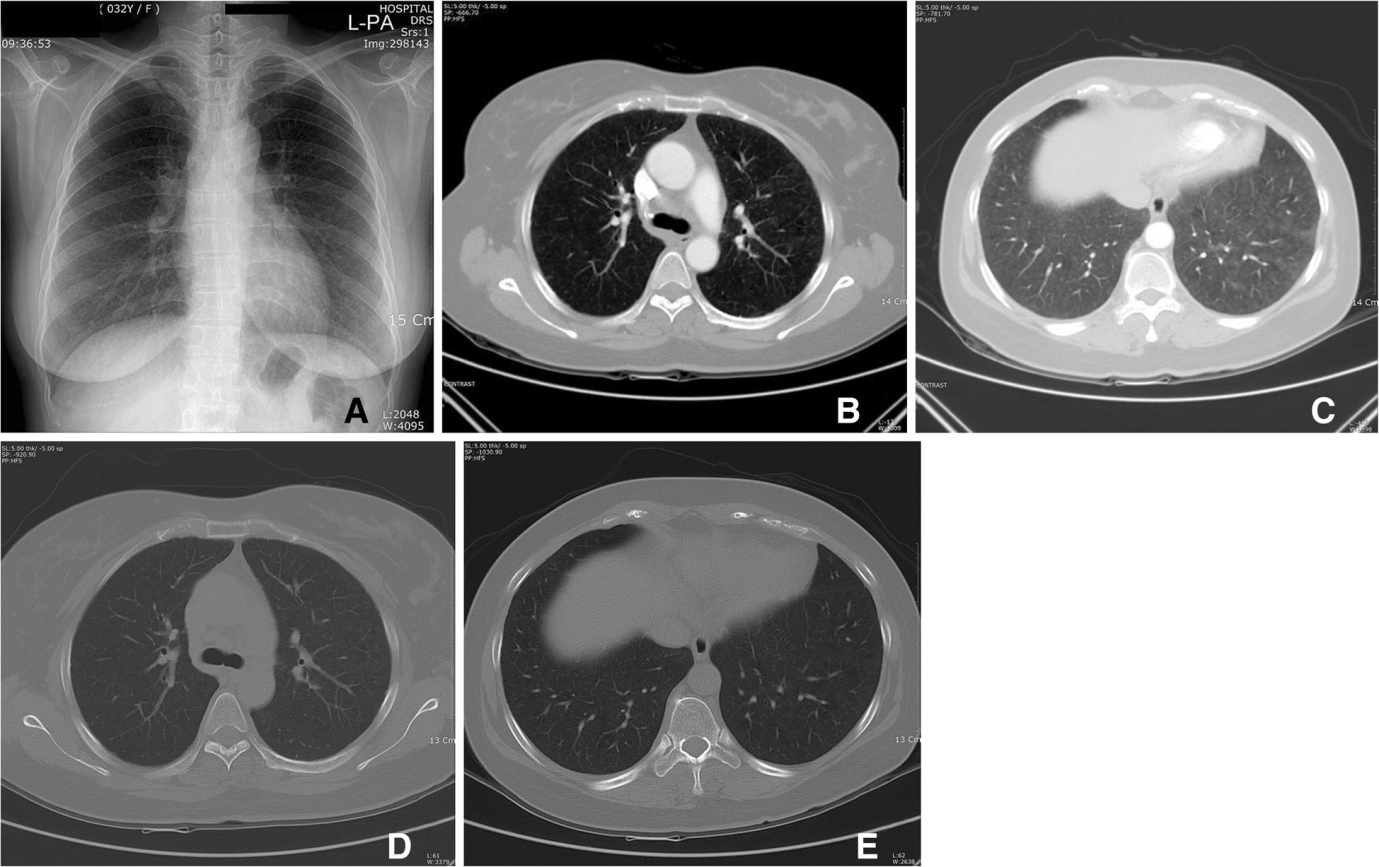
**Image findings. A**. A simple chest radiograph shows no active lesions in either lung. **B**. Chest computed tomography (CT) at the time of diagnosis shows mildly enlarged lymph nodes (arrows) in the mediastinum and both hilar and interlobar areas. **C**. The same chest CT also shows diffuse centrilobular ground-glass nodules in both lungs. **D**. Mildly enlarged lymph nodes in mediastinum and both hilar and interlobar areas can be observed on chest CT taken after being treated with medical intervention and avoiding occupational dust from the workplace. **E**. The density of diffuse centrilobular ground-glass nodules is decreased in both lungs after being treated with conservative measures.

**Figure 2:**
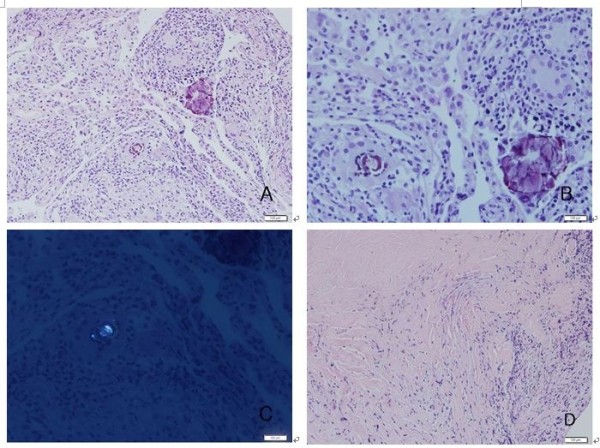
**Phathological findings. A**. On medium power, a section shows many chronic inflammatory cells with rare alveolar architecture. In the middle and upper parts of this figure, foreign body granulomas are observed (H&E, 200X). **B**. On high power, the granuloma shows several tiny, conspicuous, spiculated, and scattered metallic substances, which can be discriminated from usual calcified spherules (H&E, 400X). **C**. In the polarizing microscope, a vivid, intense birefringent metallic structure is noted in the metallic substances(400X). **D**. On medium power, the section shows dense fibrosis and rarely viable lung parenchyma (H&E, 200X).

Her blood tests including a complete blood count, electrolyte, and endocrine serum tests, and urinalysis were within normal range. A simple chest radiograph had unremarkable findings, but chest CT revealed ground glass patterns in both lungs (Figure 1C). Small nodules with emphysematous changes around the mediastinum were observed in the lower lobe of left lung, and the lymph nodes in the hilar and mediastinal areas were enlarged in both lung fields (Figure 1B, D). The BAL results showed increased white blood cell counts to 2,080 per mm^3^. The fraction of lymphocytes was found to be high, at 80%. The CD4+/CD8+ ratio of lymphocytes was 7.82, signifying an increased number of CD4+ lymphocytes. Tissue samples were acquired through transbronchial biopsy, and histologic examination of the samples, showed microcalcification, noncaseating granuloma with foreign body-type giant cells, and macrophages containing metal particles. When the metal particles were observed under a polarizing microscope, the particles were positive for birefringence (Figure 2B, C). In addition, angiotensin-converting enzyme and rheumatoid factor tests were evaluated in order to differentiate from other interstitial lung diseases such as rheumatoid nodules and sarcoidosis that have similar manifestations of mediastinal lymph node enlargement, and the tests were within normal range.

### Clinical progress

After admission, 10 mg of amlodipine besylate was given to the patient daily. Her blood pressure was controlled under 130/90 mmHg, and her symptoms subsided with the exception of an intermittent cough. Her respiratory symptoms improved significantly 2 days after she was no longer exposed the work environment. Because the number of CD4 + T cells had increased noticeably, a T-cell-mediated immune response was suspected, and 0.5 mg/kg of prednisolone was prescribed. The dyspnea showed no significant improvement, but the cough and sputum improved somewhat. She returned to her job after discharge from the hospital, and her symptoms reappeared some time after she started working. Medications were continued to reduce her symptoms. In a chest CT taken at 2 months after admission, the lung lesions observed in the previous CT had nearly disappeared (Figure 1D, E). She quit her job for 3 months, and the use of prednisolone was tapered and eventually discontinued at 5 months after initiation of the therapy. Without further exposure, the respiratory symptoms including dyspnea on exertion virtually vanished.

### Working history

The patient had worked as a dental technician since 2002. At the current work place, she had worked for 8 months, and she worked from 9 AM to 6 PM 6 days a week. As she recalled, she had witnessed that her colleagues had also suffered from intermittent cough and sputum. The company she worked for had recently been established, about a year earlier, and had 3 workers. The patient mainly worked in the trimming and ceramic build-up processes.

## Conclusions

The general work procedures of a dental technician include model marking, waxing up, investing, burn-out, casting, devesting, metal trimming, and polishing. In these processes, the workers are exposed to metal dust or fumes from casting and metal trimming, and the polishing and porcelain working rooms are where a person can be exposed to such dust. The size of dust particles to which a dental technician is exposed while performing metal trimming and polishing is about 0.3-5 μm, and studies have found that the dust can cause health problems [3]. Hong et al [4] who analyzed the main constituents of the exposure in a dental technician’s work place reported that MMA is a major particle of exposure.

### Exposure assessment of workplace

A dental technician’s work includes the following processes: polishing metal alloy acquired by casting with a diamond disk, stone point, or carbon steel tool; a washing phase such as ultrasound cleaning and smoothing surfaces by blasting with 5-μm aluminum oxide particles; a building process in which metal held in the proper position by porcelain is heated in a calcination furnace; a forming process that shapes metal alloy or resin to fit a patient’s dental and oral structures; and glossing and polishing ceramic and metal surfaces with diamond disks. Among them, the disking, forming, and polishing processes have a risk of exposure to metal and resin dust. For protection, the dental technician patient used a disposable medical mask during the manufacturing process, but no gloves were used. Polishing is a dry process in which a machine is driven manually. A dust collector is built into the working desk, and it pulls the dust under the desk. The melting work table has a hood-type fume collector installed.

Dental technician exposure to MMA or metal dust has been reported previously. In Korea, Hong et al. researched the chemical risk factors [4] for dental technicians working in the Ulsan area. In that study, the size of the dental laboratory was 6.3 workers on average, and 10 out of 14 laboratories employed fewer than 10 people. Our patient worked in a similar environment to that described by Hong et al.’s study. The patient had worked in a laboratory where 3 technicians were working, and the size of the work area was about 30-60 m^2^. Although this study did not perform direct measures of the level of exposure within the patient’s work place, the study of Hong et al. can provide indirect data on the nature of the working conditions for the present case. According to Hong et al., all 16 places were found to have manganese, cobalt, and chrome dusts as well as MMA. Since MMA induced hypersensitivity pneumonitis has been reported [5] and MMA causes pathologic condition dependent on an individual’s immune process rather than the level of MMA exposure, MMA is considered a major risk to dental technicians.

### Clinical diagnosis

HP is an interstitial disease caused by consistent and repeated exposure to dust that incites an immune reaction to the lung interstitium and alveola. Clinically, it is divided into acute, subacute, and chronic types. In the acute phase, flu like symptoms such as cough and dyspnea develop. The subacute phase proceeds for several weeks, and it is characterized by mild fever and exertional dyspnea. In the chronic type, repeated exposure to a low quantity of causative particles provokes chronic bronchitis symptoms such as exertional dyspnea, cough, and sputum, and along with the symptoms, patient may also complain of weight loss and mild fever. In the chronic type, irreversible changes, including fibrosis of lung tissue may be evident. It is imperative to consider HP in a person whose pulmonary symptoms are suspected to be related with a workplace or environment which there is a risk of organic dust exposure. In addition, when repeated episodes of symptom relapse and recovery in a person who is exposed to antigens that are known to cause HP are present, clinical work-ups are recommended [6].

The etiology of the disease is still unknown, but it is currently believed to be due to a cell-mediated and humoral immune reaction that is caused by aspirated antigen. When antibody complex is formed with aspirated antigens, the macrophages are activated. In turn, interleukin (IL)-1,-2,-6, and-8, interferon (IFN)-γ, tumor necrotizing factor (TNF)-, and macrophage inflammatory protein-1α are secreted. Initially, the number of neutrophils are increased, and later T-lymphocytes and monocytes become dominant. In the early stage, the number of CD4+ lymphocytes increases, but the number of CD8+ lymphocytes increases in the later stage, lowering the CD4+/CD8+ ratio [7]. A delayed hypersensitivity reaction is manifested by helper T1 cells including granulomas and fibrotic changes in the peribronchiolar area, and IL-12 secreted by activated macrophages is involved with differentiation from T0 to T1 cells [7]. The known causes of HP are antigens derived from animals and plants, low molecular weight chemical substances, microorganisms such as bacteria and fungi, drugs, and metal dusts. Occupationally and environmentally, light metals including cobalt and aluminum, beryllium, and MMA are also known to incite HP.

In the acute stage, hypersensitivity pneumonitis manifests fever, myalgia, cough and dyspnea starting 4 to 12 hours after exposure. With discontinuation of the exposure, the symptoms are self-limiting within a few days. Further exposure leads to the subacute stage, and several days or weeks of exposure incites symptoms such as cough and dyspnea that subside within a few weeks to months without further exposure. The protracted acute or subacute phase may develop into the chronic stage [8]. Then chest radiograph shows centrilobular nodules, ground glass opacity, airspace consolidation, and a mosaic pattern in the acute and subacute types, and in the chronic type, fibrosis and emphysema are the dominant findings [9]. BAL shows lymphocytosis with a decreased CD4+/CD8+ ratio. Interstitial lymphocyte infiltration and noncaseating granuloma are characteristic findings histologically. A simple spirometer test reveals both restrictive and obstructive patterned ventilatory dysfunction, and the pulmonary diffusing capacity is lowered. A provoking test induced by aspirating causative particles is helpful to differentiate from other interstitial lung diseases, but the test must be considered cautiously as a diagnostic tool due to the risky nature of a provoking test [9,10].

Several diagnostic criteria have been suggested. Schuyler and Cormier proposed the following symptoms to be compatible with HP: a clear exposure history or confirmation of the causative antigen from medical tests such as a blood test or BAL, a simple chest radiograph or chest computed tomography findings compatible with HP, lymphocytosis by BAL analysis, suggestive pathologic findings in a lung biopsy sample, and relapse of the disease when exposed to the causative particles again as the major diagnostic criteria, along with crackles in both lungs, decreased diffusing capacity, and dyspnea on exertion as minor diagnostic criteria. If a person fits 4 major criteria and 2 minor criteria, HP is diagnosed [11]. In this case, the patient had symptoms of HP. The patient had a definite exposure history while working as a dental technician for 10 years. Both lungs showed a diffuse ground glass pattern on chest CT, and lymphocytosis was observed from the BAL fluid analysis. Finally, interstitial lymphocyte infiltration and poorly-marginated noncaseating granuloma within the lung parenchyma was present, satisfying 5 of the major diagnostic criteria suggested by Schuyler and Cormier. The patient also complained of dyspnea on exertion, satisfying 1 minor criterion. This case had an increased CD4+/CD8+ ratio, and this finding is not compatible with the typical findings of HP, in which the ratio is lowered. However, in some cases, the CD4+/CD8+ ratio that can help differentiate between HP and sarcoidosis has been found to be as high in HP as in sarcoidosis. Thus, some reports have noted that the ratio cannot be a characteristic finding in HP [12]. Since the patient’s symptoms had improved after avoiding exposure and angiotensin-converting enzyme and rheumatoid factor were found to be normal, it is reasonable to make a diagnosis of HP in this case. Lastly, this case had been confirmed by pathology, and giant cells are considered to be findings suggesting giant cell interstitial pneumonia (GIP). Generally, confirming the presence of giant cells on histologic testing is an important pathognomonic finding in making a diagnosis of GIP when a person’s clinical manifestation is suspicious. If giant cells are identified on histologic testing, they present as many giant cell infiltrations, mostly in the interstitium and alveolar lumen. Giant cells are usually found in pulmonary tuberculosis, foreign bodies, and HP. If a low number of giant cells are present, this may suggest non-specific findings, and careful interpretation is required with counting in clinical progression and laboratory findings in making a diagnosis of a lung disease. However, in our case, the infiltration was found diffusely but minimally in both lungs, and interstitial lymphocyte infiltration and noncaseating granuloma that had an ill-defined demarcation with the lung parenchyma were other histological findings. Considering these findings, our case was more compatible with the findings of HP or dental technician’s pneumonitis in pathologic terms than with the findings of GIP. MMA, which is the presumptive particle causing the patient’s clinical manifestation, has been reported to incite immune related lung diseases such as occupational asthma and HP [13], and in an animal study, MMA was demonstrated to provoke subacute pneumonitis [14]. The etiology of HP induced by MMA is believed to be a humoral and cell-mediated immune reaction resulting in antibody complex formation. In turn, the formation activates macrophages that secrete various cytokines, creating fibrosis and granuloma, and as in our case, macrophages are characteristically observed in BAL fluid [15].

The treatment for HP is early diagnosis and avoidance of the antigen. When clinical symptoms are severe, systemic steroid hormone can be administered [8]. Prednisolone was given to the patient in our case, and the treatment improved the dyspnea and chest CT findings. She quit her job 3 months after being diagnosed with HP, and the prednisolone was tapered and eventually discontinued at 5 months after initiating the administration. Her symptoms improved significantly after she began avoiding exposure, and she no longer complained of any symptoms in her daily activities.

### Assessment of work-relatedness

Because HP has nonspecific clinical symptoms, a differential diagnosis with other diseases that cause fibrotic changes in the lung parenchyma are necessary. Therefore, to make a diagnosis of HP properly by differentiating from other lung diseases such as pulmonary tuberculosis, histoplasmosis, rheumatoid nodules, sarcoidosis, pneumoconiosis, and giant cell interstitial pneumonia, evidence of an exposure history and radiologic and laboratory tests are required. GIP, in particular, which is characterized by multinucleated giant cells, has clinical and laboratory findings similar to HP, requiring careful interpretation [16]. In our case, the patient had undergone transbronchial lung biopsy, and multinucleated giant cells were present in the sample. However, the giant cell presence was minimal but diffusely spread over the tissue, and in addition lymphocyte infiltration and noncaseating granuloma that did not have a clear border with the lung parenchyma were also observed. With these findings, the diagnosis was made as subacute HP or dental technician’s pneumoconiosis in pathologic terms. To find an association of the occupation with the patient’s condition, it is important to exclude other causes or exposures that may have derived from patient’s growth, residential environment, and hobbies. The patient had resided and been raised in an urban apartment, and she did not possess any hobby or other jobs that would suggest exposure to dust. Considering that her father worked as a white collar professional in a printing business and her mother was a homemaker, any secondary exposure from a family member could also be excluded. The patient’s symptomatic clinical progression had improved after avoiding workplace exposure. Therefore, because the patient has no other causes besides her occupational exposure and her condition had improved without further exposure to dust, we were able to determine with a high degree of certainty that her pneumonitis was associated with her occupation.

In light often clinical investigation and literature review, her condition was well-suited for making a diagnosis of HP. While evaluating her work environment, suspected MMA and light metal dusts were found. While working, it was confirmed that she was not provided with proper protection equipment, and her condition improved after quitting her job. She did not have any familial history or other jobs or hobbies that may have exposed her to the condition. These findings suggest strong work-relatedness.

This case is important that it is the second case of HP in a dental technician diagnosed in Korea. In Korea, the first case of HP in a dental technician was recognized as a occupational disease in 2005. The study has limitations in that a direct evaluation of the level of dust exposure within the workplace was not performed, and the follow-up time could have been longer. However, despite these limitations, the investigation process made in this case was valuable in that it may assist in making a diagnosis of lung parenchymal diseases in other patients and in evaluating the work-relatedness of diseases in dental technicians and those working in similar fields.

In addition, it should be noted that, the HP of a dental technician is probably work-related if the clinical signs and symptoms were subside when exposure to the workplace ends. In future, when a nonspecific case with suspicion of work-related HP is encountered, much effort will be necessary to make long-term observations of changes in the patient’s clinical condition and to perform the required examinations, including invasive ones.

### Consent

Written informed consent was obtained form the patient for the publication of this report and any accompanying images.

## Competing interests

The authors declare that they have no competing interests.

## Authors’ contributions

YH Kim and YK Chung intervewed and wrote the article. CH Kim and ES Nam supported and advised medical view. HJ Kim and YS Joo searched and assisted the related references. All of the authors read and appoved the final manuscript.
